# *N*′-[(*E*)-(4-Bromo-2-thienyl)methyl­ene]isonicotinohydrazide

**DOI:** 10.1107/S160053680903709X

**Published:** 2009-09-19

**Authors:** Zahid Shafiq, Muhammad Yaqub, M. Nawaz Tahir, Abid Hussain, M. Saeed Iqbal

**Affiliations:** a Department of Chemistry, Bahauddin Zakariya University, Multan60800, Pakistan; b Department of Physics, University of Sargodha, Sargodha, Pakistan; c Department of Chemistry, Government College University, Lahore, Pakistan

## Abstract

In title compound, C_11_H_8_BrN_3_OS, the dihedral angle between the two aromatic rings is 27.61 (14)° and the Br atom is disordered over two sites with an occupancy ratio of 0.804 (2):0.196 (2). In the crystal, the mol­ecules are linked by N—H⋯O, C—H⋯O and C—H⋯N inter­actions, resulting in chains.

## Related literature

For related structures, see: Jing *et al.* (2007 ▶); Shafiq *et al.* (2009 ▶); Wang *et al.* (2007 ▶). For graph-set notation, see: Bernstein *et al.* (1995 ▶).
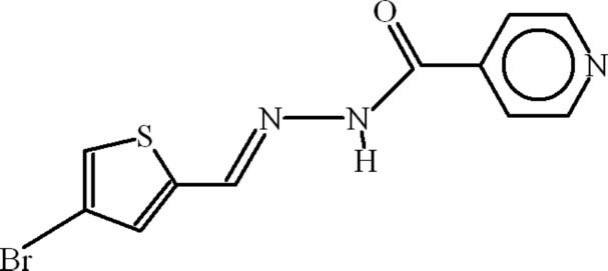


## Experimental

### 

#### Crystal data

C_11_H_8_BrN_3_OS
*M* *_r_* = 310.17
Orthorhombic, 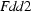

*a* = 14.3507 (6) Å
*b* = 48.732 (2) Å
*c* = 7.2115 (3) Å
*V* = 5043.3 (4) Å^3^
*Z* = 16
Mo *K*α radiation
μ = 3.41 mm^−1^
*T* = 296 K
0.26 × 0.14 × 0.12 mm

#### Data collection

Bruker Kappa APEXII CCD diffractometer
Absorption correction: multi-scan (*SADABS*; Bruker, 2005 ▶) *T*_min_ = 0.567, *T*_max_ = 0.666
12209 measured reflections
2837 independent reflections
1954 reflections with *I* > 2σ(*I*)
*R*_int_ = 0.035

#### Refinement

*R*[*F* ^2^ > 2σ(*F* ^2^)] = 0.044
*wR*(*F* ^2^) = 0.113
*S* = 1.04
2837 reflections
158 parameters
2 restraints
H-atom parameters constrained
Δρ_max_ = 0.56 e Å^−3^
Δρ_min_ = −0.48 e Å^−3^
Absolute structure: Flack (1983 ▶), 1205 Friedal Pairs
Flack parameter: −0.002 (13)

### 

Data collection: *APEX2* (Bruker, 2007 ▶); cell refinement: *SAINT* (Bruker, 2007 ▶); data reduction: *SAINT*; program(s) used to solve structure: *SHELXS97* (Sheldrick, 2008 ▶); program(s) used to refine structure: *SHELXL97* (Sheldrick, 2008 ▶); molecular graphics: *ORTEP-3 for Windows* (Farrugia, 1997 ▶) and *PLATON* (Spek, 2009 ▶); software used to prepare material for publication: *WinGX* (Farrugia, 1999 ▶) and *PLATON*.

## Supplementary Material

Crystal structure: contains datablocks global, I. DOI: 10.1107/S160053680903709X/hb5101sup1.cif

Structure factors: contains datablocks I. DOI: 10.1107/S160053680903709X/hb5101Isup2.hkl

Additional supplementary materials:  crystallographic information; 3D view; checkCIF report

## Figures and Tables

**Table 1:** Hydrogen-bond geometry (Å, °)

*D*—H⋯*A*	*D*—H	H⋯*A*	*D*⋯*A*	*D*—H⋯*A*
N2—H2⋯O1^i^	0.86	2.08	2.920 (4)	165
C7—H7⋯O1^i^	0.93	2.52	3.318 (5)	144
C11—H11⋯N1^ii^	0.93	2.60	3.277 (7)	130

Symmetry codes: (i) 
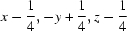
; (ii) 
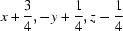
.

**Fig. 1.**
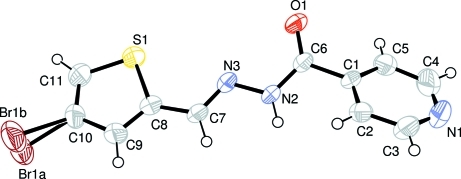
View of (I) with displacement ellipsoids drawn at the 50% probability level. H-atoms are shown by small circles of arbitrary radius.

**Fig. 2.**
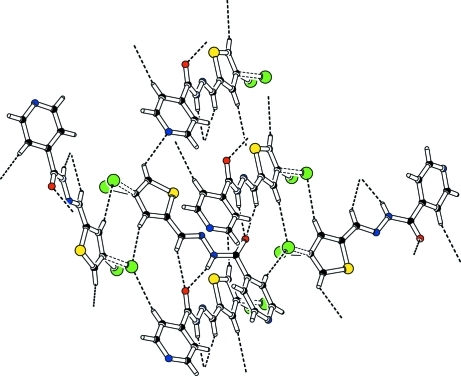
The partial packing of (I) which shows that molecules form polymeric chains with ring motifs due to H-bondings.

## supplementary crystallographic information

### Comment

In continuation of synthesizing various derivatives of 4-bromothiophene-2-carbaldehyde (Shafiq *et al.*, 2009), the title compound (I, Fig. 1), has been prepared. The metal complexes of (I) have been prepared and the biological studies of all the compounds are in progress.

The crystal structure of (II) *N*'-((thiophen-3-yl)methylene)isonicotinohydrazide (Jing *et al.*, 2007) and (III) (*E*)-*N*'-((5-methylthiophen-2-yl)methylene)isonicotinohydrazide (Wang *et al.*, 2007) have been published. The title compound (I) differs from both due to substitution moiety of bromo.

We have recently reported the crystal structure of (IV) *N*'-[(*E*)-(4-bromothiophen-2-yl)methylidene]benzohydrazide (Shafiq *et al.*, 2009). Due to change of benzene ring (IV) with the pyridine (I) ring, the crystal structure has been substantially changed. The title compound crystalizes with single molecule, whereas in (IV) there are two molecules along with fractional quantity of water. In (I), the dihedral angle between two aromatic rings A (C1—C3/N1/C4/C5) and B (C8—C11/S1) is 27.61 (14)°. The molecules of present compound are stabilized in the form of polymeric chains extending along the diagonal of crystallographic *ac*-plane. The list of strong H-bondings is given in Table 1. Due to the heterocyclic rings, the Br-Atom is disordered over two sites with occupancy ratio of 0.804:0.196. There exist *R*_2_^1^(6) ring motif (Bernstein *et al.*, 1995) due to intermolecular H-bonding of type C—H···O and N—H···O (Fig. 2).

### Experimental

To a hot stirred solution of isoniazid (1.37 g, 0.01 mol) in ethanol (15 ml) was added 4-bromothiophene-2-carbaldehyde (1.91 g, 0.01 mol). The resultant mixture was then heated under reflux. After an hour precipitates were formed. The reaction mixture was further heated about 30 min for the completion of the reaction which was monitored through TLC. Then it was allowed to cool to room temperature, filtered and washed with hot ethanol. The crude material was recrystallized in (1:3 *v*/*v*) 1,4-dioxan:ethanol, to afford light yellow needles of (I).

### Refinement

A large Fourier difference peak close to the Br-atom and higher values of its thermal parameters indicated the presence of disorder. The two parts of Br-atom were refined with equal ainisotropic displacement parameters (EADP). All other efforts like *DFIX* were utilized but the C10—Br1B bond could not be shortened.

The H-atoms were positioned geometrically with N—H = 0.86, C—H = 0.93 Å for aromatic like H atoms and constrained to ride on their parent atoms, with *U*_iso_(H) = xUeq(C, N), where *x* = 1.2 for all H atoms.

### Crystal data

**Table:**

C_11_H_8_BrN_3_OS	*F*(000) = 2464
*M**_r_* = 310.17	*D*_x_ = 1.634 Mg m^−^^3^
Orthorhombic, *F**d**d*2	Mo *K*α radiation, λ = 0.71073 Å
Hall symbol: F 2 -2d	Cell parameters from 2837 reflections
*a* = 14.3507 (6) Å	θ = 3.0–27.9°
*b* = 48.732 (2) Å	µ = 3.41 mm^−^^1^
*c* = 7.2115 (3) Å	*T* = 296 K
*V* = 5043.3 (4) Å^3^	Cut needle, light yellow
*Z* = 16	0.26 × 0.14 × 0.12 mm

### Data collection

**Table:**

Bruker Kappa APEXII CCD diffractometer	2837 independent reflections
Radiation source: fine-focus sealed tube	1954 reflections with *I* > 2σ(*I*)
graphite	*R*_int_ = 0.035
Detector resolution: 7.50 pixels mm^-1^	θ_max_ = 27.9°, θ_min_ = 3.0°
ω scans	*h* = −18→18
Absorption correction: multi-scan (*SADABS*; Bruker, 2005)	*k* = −64→63
*T*_min_ = 0.567, *T*_max_ = 0.666	*l* = −9→9
12209 measured reflections	

### Refinement

**Table:**

Refinement on *F*^2^	Secondary atom site location: difference Fourier map
Least-squares matrix: full	Hydrogen site location: inferred from neighbouring sites
*R*[*F*^2^ > 2σ(*F*^2^)] = 0.044	H-atom parameters constrained
*wR*(*F*^2^) = 0.113	*w* = 1/[σ^2^(*F*_o_^2^) + (0.0415*P*)^2^ + 9.3783*P*] where *P* = (*F*_o_^2^ + 2*F*_c_^2^)/3
*S* = 1.04	(Δ/σ)_max_ < 0.001
2837 reflections	Δρ_max_ = 0.56 e Å^−^^3^
158 parameters	Δρ_min_ = −0.48 e Å^−^^3^
2 restraints	Absolute structure: Flack (1983), 1205 Friedal Pairs
Primary atom site location: structure-invariant direct methods	Flack parameter: −0.002 (13)

### Special details

**Table:**

Geometry. Bond distances, angles *etc*. have been calculated using the rounded fractional coordinates. All su's are estimated from the variances of the (full) variance-covariance matrix. The cell e.s.d.'s are taken into account in the estimation of distances, angles and torsion angles
Refinement. Refinement of *F*^2^ against ALL reflections. The weighted *R*-factor *wR* and goodness of fit *S* are based on *F*^2^, conventional *R*-factors *R* are based on *F*, with *F* set to zero for negative *F*^2^. The threshold expression of *F*^2^ > σ(*F*^2^) is used only for calculating *R*-factors(gt) *etc*. and is not relevant to the choice of reflections for refinement. *R*-factors based on *F*^2^ are statistically about twice as large as those based on *F*, and *R*- factors based on ALL data will be even larger.

### Fractional atomic coordinates and isotropic or equivalent isotropic displacement parameters (Å^2^)

**Table:**

	*x*	*y*	*z*	*U*_iso_*/*U*_eq_	Occ. (<1)
Br1A	0.42406 (6)	0.25903 (2)	−0.16312 (17)	0.0946 (5)	0.804 (2)
Br1B	0.4270 (3)	0.26462 (9)	−0.0262 (10)	0.0946 (5)	0.196 (2)
S1	0.44553 (8)	0.17467 (2)	0.0252 (2)	0.0549 (4)	
O1	0.27773 (19)	0.09307 (6)	0.2899 (5)	0.0505 (10)	
N1	−0.0545 (3)	0.06098 (14)	0.3270 (10)	0.099 (3)	
N2	0.1935 (2)	0.12740 (7)	0.1634 (5)	0.0406 (11)	
N3	0.2717 (2)	0.14261 (7)	0.1259 (5)	0.0424 (11)	
C1	0.1129 (3)	0.08803 (8)	0.2787 (6)	0.0418 (14)	
C2	0.0333 (3)	0.10184 (10)	0.3343 (7)	0.0527 (16)	
C3	−0.0467 (3)	0.08712 (15)	0.3572 (10)	0.080 (3)	
C4	0.0234 (5)	0.04764 (13)	0.2750 (10)	0.092 (3)	
C5	0.1084 (4)	0.06041 (10)	0.2517 (8)	0.0620 (19)	
C6	0.2039 (3)	0.10290 (8)	0.2448 (6)	0.0382 (14)	
C7	0.2561 (3)	0.16678 (9)	0.0670 (7)	0.0460 (16)	
C8	0.3315 (3)	0.18499 (9)	0.0213 (7)	0.0470 (14)	
C9	0.3246 (3)	0.21142 (11)	−0.0287 (9)	0.068 (2)	
C10	0.4113 (3)	0.22347 (9)	−0.0687 (9)	0.0643 (19)	
C11	0.4832 (3)	0.20625 (11)	−0.0432 (9)	0.0653 (18)	
H2	0.13903	0.13346	0.13520	0.0485*	
H2A	0.03442	0.12066	0.35548	0.0632*	
H3	−0.09935	0.09650	0.39733	0.0962*	
H4	0.01970	0.02885	0.25376	0.1106*	
H5	0.16110	0.05044	0.21847	0.0746*	
H7	0.19500	0.17278	0.05327	0.0554*	
H9	0.26831	0.22080	−0.03612	0.0813*	
H11	0.54538	0.21094	−0.06045	0.0783*	

### Atomic displacement parameters (Å^2^)

**Table:**

	*U*^11^	*U*^22^	*U*^33^	*U*^12^	*U*^13^	*U*^23^
Br1A	0.0709 (4)	0.0603 (5)	0.1526 (14)	−0.0081 (3)	0.0142 (7)	0.0410 (7)
Br1B	0.0709 (4)	0.0603 (5)	0.1526 (14)	−0.0081 (3)	0.0142 (7)	0.0410 (7)
S1	0.0358 (5)	0.0516 (7)	0.0773 (9)	0.0035 (5)	−0.0020 (6)	0.0081 (6)
O1	0.0291 (14)	0.0495 (17)	0.073 (2)	0.0004 (12)	−0.0118 (16)	0.0034 (17)
N1	0.056 (3)	0.111 (5)	0.130 (5)	−0.041 (3)	−0.031 (3)	0.053 (4)
N2	0.0250 (16)	0.0437 (19)	0.053 (2)	−0.0055 (14)	−0.0080 (15)	0.0056 (16)
N3	0.0281 (16)	0.049 (2)	0.050 (2)	−0.0061 (15)	−0.0043 (15)	0.0029 (16)
C1	0.0315 (19)	0.048 (2)	0.046 (3)	−0.0076 (17)	−0.0167 (19)	0.007 (2)
C2	0.037 (2)	0.062 (3)	0.059 (3)	0.0025 (19)	−0.002 (2)	0.022 (2)
C3	0.039 (3)	0.108 (5)	0.094 (5)	−0.011 (3)	−0.012 (3)	0.042 (4)
C4	0.091 (5)	0.068 (4)	0.118 (6)	−0.032 (3)	−0.041 (4)	0.030 (4)
C5	0.062 (3)	0.046 (3)	0.078 (4)	−0.007 (2)	−0.026 (3)	0.012 (3)
C6	0.0276 (19)	0.043 (2)	0.044 (3)	−0.0029 (16)	−0.0040 (17)	−0.0027 (19)
C7	0.029 (2)	0.056 (3)	0.053 (3)	−0.0051 (18)	−0.0089 (19)	0.011 (2)
C8	0.034 (2)	0.049 (2)	0.058 (3)	0.0006 (17)	−0.008 (2)	0.006 (2)
C9	0.039 (2)	0.057 (3)	0.107 (5)	0.002 (2)	−0.008 (3)	0.026 (3)
C10	0.045 (3)	0.046 (3)	0.102 (4)	−0.007 (2)	−0.001 (3)	0.017 (3)
C11	0.035 (2)	0.063 (3)	0.098 (4)	−0.003 (2)	0.000 (3)	0.015 (3)

### Geometric parameters (Å, °)

**Table:**

Br1A—C10	1.871 (5)	C2—C3	1.364 (7)
Br1B—C10	2.041 (6)	C4—C5	1.380 (9)
S1—C8	1.712 (4)	C7—C8	1.438 (6)
S1—C11	1.704 (5)	C8—C9	1.341 (7)
O1—C6	1.207 (5)	C9—C10	1.406 (6)
N1—C3	1.297 (10)	C10—C11	1.343 (6)
N1—C4	1.347 (9)	C2—H2A	0.9300
N2—N3	1.372 (4)	C3—H3	0.9300
N2—C6	1.339 (5)	C4—H4	0.9300
N3—C7	1.272 (6)	C5—H5	0.9300
N2—H2	0.8600	C7—H7	0.9300
C1—C2	1.385 (6)	C9—H9	0.9300
C1—C5	1.362 (6)	C11—H11	0.9300
C1—C6	1.513 (6)		
			
C8—S1—C11	91.9 (2)	Br1A—C10—C11	123.6 (4)
C3—N1—C4	116.7 (5)	Br1B—C10—C9	118.5 (4)
N3—N2—C6	118.5 (3)	C9—C10—C11	113.0 (4)
N2—N3—C7	115.0 (3)	Br1B—C10—C11	120.6 (4)
C6—N2—H2	121.00	Br1A—C10—C9	123.3 (4)
N3—N2—H2	121.00	S1—C11—C10	111.1 (3)
C2—C1—C6	121.7 (4)	C1—C2—H2A	121.00
C5—C1—C6	119.4 (4)	C3—C2—H2A	121.00
C2—C1—C5	118.9 (4)	N1—C3—H3	118.00
C1—C2—C3	118.3 (5)	C2—C3—H3	118.00
N1—C3—C2	124.7 (5)	N1—C4—H4	118.00
N1—C4—C5	123.4 (6)	C5—C4—H4	118.00
C1—C5—C4	118.1 (5)	C1—C5—H5	121.00
N2—C6—C1	113.7 (3)	C4—C5—H5	121.00
O1—C6—C1	121.6 (4)	N3—C7—H7	120.00
O1—C6—N2	124.7 (4)	C8—C7—H7	119.00
N3—C7—C8	121.0 (4)	C8—C9—H9	123.00
C7—C8—C9	126.8 (4)	C10—C9—H9	123.00
S1—C8—C7	122.3 (3)	S1—C11—H11	124.00
S1—C8—C9	110.9 (3)	C10—C11—H11	124.00
C8—C9—C10	113.0 (4)		
			
C11—S1—C8—C7	179.5 (5)	C2—C1—C6—N2	−38.5 (6)
C11—S1—C8—C9	−0.5 (5)	C5—C1—C6—O1	−39.9 (7)
C8—S1—C11—C10	−0.5 (5)	C5—C1—C6—N2	140.6 (5)
C4—N1—C3—C2	2.2 (11)	C1—C2—C3—N1	−1.5 (10)
C3—N1—C4—C5	−0.6 (11)	N1—C4—C5—C1	−1.6 (10)
C6—N2—N3—C7	−172.8 (4)	N3—C7—C8—S1	5.3 (7)
N3—N2—C6—O1	0.6 (6)	N3—C7—C8—C9	−174.7 (5)
N3—N2—C6—C1	−179.9 (3)	S1—C8—C9—C10	1.4 (7)
N2—N3—C7—C8	−179.7 (4)	C7—C8—C9—C10	−178.6 (5)
C5—C1—C2—C3	−0.9 (8)	C8—C9—C10—Br1A	174.2 (4)
C6—C1—C2—C3	178.3 (5)	C8—C9—C10—C11	−1.9 (8)
C2—C1—C5—C4	2.2 (8)	Br1A—C10—C11—S1	−174.6 (3)
C6—C1—C5—C4	−176.9 (5)	C9—C10—C11—S1	1.4 (7)
C2—C1—C6—O1	141.0 (5)		

### Hydrogen-bond geometry (Å, °)

**Table:**

*D*—H···*A*	*D*—H	H···*A*	*D*···*A*	*D*—H···*A*
N2—H2···O1^i^	0.86	2.08	2.920 (4)	165
C7—H7···O1^i^	0.93	2.52	3.318 (5)	144
C11—H11···N1^ii^	0.93	2.60	3.277 (7)	130

Symmetry codes: (i) *x*−1/4, −*y*+1/4, *z*−1/4; (ii) *x*+3/4, −*y*+1/4, *z*−1/4.
